# Novel Antioxidants for Animal Nutrition

**DOI:** 10.3390/antiox13040438

**Published:** 2024-04-07

**Authors:** Luciana Rossi, Matteo Dell’Anno

**Affiliations:** Department of Veterinary Medicine and Animal Sciences—DIVAS, University of Milan, 29600 Lodi, Italy; luciana.rossi@unimi.it

In recent years, the importance of nutrition has notably escalated, with antioxidants emerging as crucial ingredients in the formulation of functional diets pivotal for promoting animal health and preventing diseases. Antioxidants can be obtained from a variety of natural sources or can be chemically synthesized in laboratory settings. Oxidative balance refers to the equilibrium between oxidants and antioxidants, and it is crucial for overall health. On the contrary, oxidative stress, resulting from a disparity favoring oxidants, can lead to cellular damage and contribute to the development of pathological states. The role of dietary antioxidants has also gained renewed interest in cancer research.

Antioxidants offer multifaceted benefits in animal diets, including supporting animal health, reducing disease incidence, and ultimately contributing to the reduction of antibiotics use in livestock. By promoting overall health and bolstering the immune system, antioxidants help to decrease the need for antimicrobials. Additionally, sustainable farming practices encourage the inclusion of antioxidants, aligning with the efforts to mitigate antimicrobial resistance, and supporting public health and food safety initiatives.

The bioaccessibility of antioxidant compounds can be influenced by various factors that are not only related to the differences in the digestive systems of ruminants and monogastrics, but also by the enzymatic portfolio, feed composition, microbiota contributors, and many other species-specific variables. Over the recent decade, a wide range of natural extracts and compounds have been placed on the market as functional ingredients or additives. Nevertheless, their heterogeneity and varying dosages have resulted in contradictory findings in the literature, necessitating further in-depth investigations to thoroughly characterize each antioxidant for optimal inclusion in animal feed.

Beslo et al. (Contribution 1) reviewed the use of plant polyphenols in animal nutrition, proposing their use in animal nutrition for a sustainable approach and underlining the importance of understanding the intracellular mechanisms of polyphenols’ antioxidant activity to maximize their potential. Cell models offer a valuable tool for evaluating antioxidants by providing a controlled experimental system to study their protective effects against oxidative stress and their potential applications in promoting cellular health and mitigating intestinal disease processes. Cell models also contribute to the reduction of using animals in research by providing an alternative approach to study biological processes. In this context, the contribution of Xu et al. (Contribution 2) represents an intriguing approach focused on the IPEC-J2 cell model to evaluate stevioside, a natural sweetener. It registered protective effects on intestinal cells under induced oxidative stress, indicating its potential application in promoting gut health and mitigating inflammation and apoptosis. Dabrowska et al. (Contribution 3) evaluated the chlorogenic acid derived from green coffee extract on peripheral blood mononuclear cells from racehorses’ blood, suggesting its potential as a natural supplement for improving immune function and oxidative stress management in equine species.

Antioxidant supplementation in animal nutrition could enhance animal welfare, the quality of products, and reduce stress and the use of antimicrobials, which is in line with One Health principles. This is particularly relevant in young animals due to their susceptibility to stressors and immune system immaturity, highlighting the importance of proactive measures in promoting their health and welfare. In particular, piglets face numerous challenges during the weaning period that can compromise their immune system, increasing the high risk of pathogenic invasions (Contribution 4). Cai et al. (Contribution 5) investigated the protective role of silybin, derived from *Silybum marianum*, against a hepatic oxidative injury induced by paraquat in weaned piglets. Results demonstrated that a dietary supplementation of 400 mg/kg silybin alleviated liver damage by enhancing antioxidant capacity, inhibiting inflammation, and improving mitochondrial function. Pastorelli et al. (Contribution 6) evaluated the partial replacement of synthetic vitamin E by natural polyphenols in post-weaning piglets, revealing encouraging results in maintaining growth performance, antioxidant status, and immunity. Their findings suggest that the replacement of an equivalent of 87.9 mg/kg vitamin E with polyphenols extracted from different plants could be viable alternatives to conventional vitamin E commonly used in animal nutrition. Furthermore, the addition of 3% thyme to diets for fattening pigs showed positive effects on redox status and lipid metabolism, highlighting the potential of this plant extract as a functional feed additive for adult pigs (Contribution 7).

Furthermore, Laviano et al. (Contribution 8) assessed the effect of hydroxytyrosol supplementation in the colostrum and milk in Iberian sows during late pregnancy. Their study revealed that hydroxytyrosol supplementation increased polyunsaturated fatty acids in the colostrum and exhibited a lower desaturase capacity in 20-day milk. These modifications in milk lipid composition could potentially benefit the oxidative status and gut health of piglets during the early stages of life.

In broilers, selenium nanoparticles and organic selenium supplementation (0.5 mg/kg) showed positive effects in the physico-chemical quality of the breast, health status, and the antioxidant potential in muscle and liver (Contribution 9). Particularly, the nano-sized Se confirmed the protective activity against mitochondrial damage in hepatocytes without showing any toxic effects. The use of myricetin, a natural flavonoid, supplemented at 600 mg/kg in broilers showed a significant improvement in growth performance and antioxidant capacity of meat, and reduced the interleukin relative expression, severity of *Eimeria* spp. lesions, and oocyst shedding in challenged broiler chickens (Contribution 10).

In calves, in-milk dietary supplementation of 6 g/day of chestnut and quebracho tannins reduced the occurrence of neonatal calf diarrhea without affecting the protein digestibility of milk. Thus, this confirmed the role of natural polyphenols in the reduction of pathology occurrence and antibiotic treatments (Contribution 11).

In dairy ewes, a mixture of natural antioxidants derived from thyme, anise, and olive improved milk yield, oxidative stability, and somatic cell count, without influencing the milk composition. This underscored the potential of plant bioactive compounds to enhance milk quality and udder health (Contribution 12). Dalaka et al. (Contribution 13) investigated the antioxidant capacity of sweet whey derived from cheeses obtained from bovine, ovine, caprine, or a mixture of ovine/caprine milk before and after in vitro digestion. Particularly, ovine sweet whey exhibited higher antioxidant activity in both non-digested and digested cheese, suggesting ovine whey as a valuable source of antioxidants for both human and animal nutrition.

Mavrommatis et al. (Contribution 14) focused on microalgae as renewable and sustainable sources of bioactive compounds. The antioxidant activity of microalgae significantly varies between species and depends on growth conditions. The use of microalgae in animal nutrition revealed variable findings that were highly dependent on the composition of species and their percentage of inclusion. The use of carbohydrate-active enzymes can increase nutrient bioavailability because of recalcitrant microalgae cell wall degradation, making it a promising strategy for monogastric nutrition and for improving livestock productivity. The main limitations of microalgae lie in their cost-effectiveness and their cultivation technology, which should be improved by reducing production costs, thus increasing profitability.

Collectively, the published studies underlined the importance of harnessing the potential of natural antioxidants in animal nutrition. By exploring a wide array of bioactive compounds and their mechanisms of action, researchers are plotting the way for sustainable approaches in livestock farming. As we strive for a more holistic and ethical approach in livestock production, the supplementation of natural antioxidants into animal feed represents a promising frontier for enhancing health, welfare, and productivity in livestock.

